# Identification and validation of hub genes for diabetic retinopathy

**DOI:** 10.7717/peerj.12126

**Published:** 2021-09-13

**Authors:** Li Peng, Wei Ma, Qing Xie, Baihua Chen

**Affiliations:** 1 Department of Ophthalmology, The Second Xiangya Hospital of Central South University, Changsha, Hunan, China; 2 Department of Ophthalmology, Central South University Xiangya School of Medicine Affiliated Haikou Hospital, Haikou, Hainan, China

**Keywords:** Diabetic retinopathy, Weighted gene co-expression network analysis, Key genes, SLC25A33, NDUFS1

## Abstract

**Background:**

Diabetic retinopathy (DR) is characterized by a gradually progressive alteration in the retinal microvasculature that leads to middle-aged adult acquired persistent blindness. Limited research has been conducted on DR pathogenesis at the gene level. Thus, we aimed to reveal novel key genes that might be associated with DR formation via a bioinformatics analysis.

**Methods:**

The GSE53257 dataset from the Gene Expression Omnibus was downloaded for gene co-expression analysis. We identified significant gene modules via the Weighted Gene Co-expression Network Analysis, which was conducted by the Protein-Protein Interaction (PPI) Network via Cytoscape and from this we screened for key genes and gene sets for particular functional and pathway-specific enrichments. The hub gene expression was verified by real-time PCR in DR rats modeling and an external database.

**Results:**

Two significant gene modules were identified. Significant key genes were predominantly associated with mitochondrial function, fatty acid oxidation and oxidative stress. Among all key genes analyzed, six up-regulated genes (*i.e.*, SLC25A33, NDUFS1, MRPS23, CYB5R1, MECR, and MRPL15) were highly and significantly relevant in the context of DR formation. The PCR results showed that SLC25A33 and NDUFS1 expression were increased in DR rats modeling group.

**Conclusion:**

Gene co-expression network analysis highlights the importance of mitochondria and oxidative stress in the pathophysiology of DR. DR co-expressing gene module was constructed and key genes were identified, and both SLC25A33 and NDUFS1 may serve as potential biomarker and therapeutic target for DR.

## Introduction

Diabetic retinopathy (DR) is one of the most adverse complications of diabetes, which has emerged as the most common cause of visual impairment and irreversible blindness among working adults and middle-aged people (Heng et al., 2013; Mishra et al., 2016; Platania et al., 2018). Globally, an estimated 415 million people with diabetes in 2015, the patient number expected to rise up to 642 million by the year 2040. The estimated annual incidence of diabetic retinopathy ranged from 2.2% to 12.7% and progression from 3.4% to 12.3% (Sabanayagam et al., 2019). Although therapeutic approaches for DR have improved, such as photocoagulation with an argon laser, and intravitreal injections of anti- Vascular Endothelial Growth Factor (VEGF) therapy, the incidence of visual impairment in patients with proliferative diabetic retinopathy is not lower and treatment of DR remains challenging (Khan et al., 2020; Rodríguez et al., 2019). Increasing evidence shows that genetic features, oxidative stress, mitochondria dysfunction, lipid/lipoprotein-associated, pro-inflammatory, and advanced glycation end-products (AGEs), as well as environmental factors contributed to the etiology and development of DR (Jenkins et al., 2015). However, the exact pathogenesis of DR is complicated and remains largely unclear (Heng et al., 2013; Thebeau et al., 2020).

With the progress of genome-wide research, novel biomarkers include those associated with inflammation and angiogenesis that are known to play important roles in the development of novel therapeutics (Jenkins et al., 2015). Recently, circulating miRNAs as non-invasive biomarker to identify and monitored of diabetes microvascular complications has been studied (Greco et al., 2020). Previous studies have suggested that miRNAs offer insights into the pathophysiological states of DR (Mammadzada et al., 2019). Accumulating studies have highlighted critical roles for miRNAs in diabetic retinopathy, which may offer new targets for early detection and therapeutic intervention of diabetic retinopathy (Zhang et al., 2017). A thorough investigation of the molecular mechanisms of DR is critical. However, the value of their clinical application is currently limited and requires further study. Simultaneously, some associated genes have not been reported and the gene networks thought to be connected with the etiology of DR have not been clearly defined.

The aim of this paper is to further elucidate the interplay of genetic biomarkers and enriched signaling pathways associated with the pathogenesis of DR, clarifying potential genes and biological pathways that may contribute to the discovery of new and valuable targets for the treatment aimed at decreasing vision loss in DR patients.

The Gene Expression Omnibus is a public and freely available database to obtain gene expression datasets with valuable information and new insights into the molecular pathogenesis of DR. WGCNA clustering criteria play a significant biological role and much research effort has been devoted to understanding the molecular mechanisms of many diseases (You et al., 2018). A comprehensive integration of gene co-expression network in DR is still rare for the present. Therefore, it is necessary to use systems biology tools to gather datasets to forecasted the functional gene networks and obtain the stable and credible results, which may be clusters of genes with biological implications having important roles in the pathogenesis and development of DR.

## Materials and Methods

### Material and data

We downloaded the datasets selected in this study from the Gene Expression Omnibus (GEO) (https://www.ncbi.nlm.nih.gov/geo/query/acc.cgi?acc=GSE53257). We acquired the original gene expression profile from the GSE53257 dataset that was provided by Govindarajan, which included 16 human retina samples that were classified into three study groups as follows: (1) retina of patients with diabetic retinopathy; (2) retina of diabetic patients with no signs of diabetic retinopathy; and (3) retina of control patients without diabetes that were extracted from cadaveric eyes. This classification described the preparation of samples in their study. Then, microarray analyses and experiments were completed, following which, the Agilent custom algorithm was used to design the probe sets that were printed on the GPL18056 platform. The robust multiarray average algorithm (Sahlabadi et al., 2018) was used to perform quartile data standardization of the downloaded data and background correction. We filtered the lack of corresponding gene symbols for the probes, and reserved the maximum values of the gene symbols using multiple probes. All data were processed with the Limma package of R software (Version 3.5.3) as described previously (Han et al., 2020).

### Weighted gene co-expression network analysis

After raw data preprocessing, the weighted gene co-expression network was constructed by the WGCNA package of R (version 1.69) to identify the importance of genes and associated modules in this study as described previously (Han et al., 2020). The WGCNA procedure calculated a Pearson correlation matrix for all genes in a pairwise manner then a correlation matrix was calculated. The soft threshold (power) value was set at “8”. The matrix was converted into an adjacency matrix by raising all values to a power “ *β*” from the correlation matrix. Average linkage hierarchical clustering was then created to categorize modules of closely interrelated genes. According to the topological overlap matrix dissimilarity function was signed as a TOM-Type, and network inter-connectedness was performed by calculating the topologic overlap. On being built on the 1-TOM in terms of their connection strengths, the genes were grouped by average hierarchical clustering, which was measured by means of the hclust function. Modules were referred to as groups of exceedingly co-expressed genes, which usually consisted of more than 30 genes. After relating modules to clinical traits, modules with the highest correlation coefficient were choosed for subsequent analysis.

### Protein–protein interaction (PPI) network construction and analysis

To detect the relationship between genes at the protein level and to identify the key genes that were included in modules, the Cytoscape (version 3.4.0) software was applied in order to search experiment-validated PPIs amongst common selected module genes and visualize networks. Next, topologic properties of the computing, the degree, and the “betweenness” of the distribution network was analyzed using the cytoHubba app in two module PPIs.

### Gene function analysis and functional enrichment analysis

Currently, Gene Ontology (GO) is the most widely acknowledged gene function knowledge base. The cluster profiler package (Yu et al., 2012) was used in this study to evaluate gene function profiles and gene clusters with the aim of recognizing biological functions of the primary gene in the modules. According to each given gene list from selected modules, we could implement pathway and process enrichment analyses *via* the following ontology sources: GO for biological processes, GO for molecular functions, and GO for cellular components. Herein, 10 biological processes with a *p*-value of each module were listed, which reflected functional characteristics of the modules.

### Validation of the key genes

Validation of key gens was divided in two parts. One part is that we used the diabetic rats model to validate the candidate genes through quantitative reverse transcription (q-PCR) analysis. Another part is an external databases validation.

### Validation of the DR model in rats

#### Animals

Fourteen healthy 10-week-old male Sprague Dawley (SD) rats, average weighing between 300–400 g with clear ocular media and no ocular fundal lesions were purchased from Changsha Tianqin Biotechnology Co., Ltd. (Changsha, Hunan, China). The SD rats and their feeds were tested physically and chemically by the Center for Disease Control and Prevention of Hunan Province (sample acceptance Nos. 2017DW041, 2017DS009). All rats were housed in a pathogen-free facility under controlled environment with ad libitum access to water and food (temperature 18–25 °C; humidity 50%–70%; and light cycle 12-h light/12-h dark).

### Diabetes model and experimental grouping

SD rats were randomly divided into two groups, including diabetic rats and control group. After fasting for 12 h, rat models of diabetes were established by intraperitoneal injection of 60 mg/kg 1% streptozotocin solution (STZ) (Portillo et al., 2017). Moreover, 3 day after STZ injection blood glucose (BG) in caudal venous of rat were detected, and BG levels of >16.7 mmol/L were considered as a success model. Rats with blood glucose levels below 16.7 mmol/l were excluded from our study. After successful modeling, body weights and fasting blood glucose levels of rats were measured once every 4 weeks. The rats were sacrificed 3 months after the model was established. To achieve loss of consciousness and death with a minimal pain, suffering and distress to animals, all rats were euthanized by rapid cervical dislocation. Then, the rat eyeballs were obtained and rat retinal issues were collected. There were no surviving rats at the end of experiment. This study complied with the Chinese guidelines of the Experimental Animals and was approved by the Ethical Committee of Central South University Xiangya School of Medicine Affiliated Haikou Hospital (SC20170103).

### Quantitative PCR

Total RNA extraction from rat retina tissues with TRIzol method, mRNA reverse transcribed to cDNA and real-time quantitative PCR (qPCR) were performed according with the manufacturer’s instructions. The primer sequence information was shown in Table S1. The primers were synthesized by Shanghai Biotechnology (Shanghai, China). qPCR was conducted in a total reaction volume of 30 µl, including 2 µl of template cDNA, 1 uL each of Primer R and Primer F, 15 uL of 2X SYBGREEN PCR Master Mix (Kangwei Century Co. LTD, Beijing, China) and 11 uL of ddH2O. Then, 40 cycles of an amplification and quantification program (95 °C for 15 s and 60 °C for 30 s) were carried out and melt curve analysis was performed.

### The conservation analysis of SLC25A33 and NDUFS1 in human, mouse and rat

The conservation analysis of SLC25A33 and NDUFS1 were made *via* DNAMAN software (https://www.lynnon.com/dnaman.html) and National Center for Biology Information (NCBI) database in human, mouse, and rat.

### Validation of the external dataset

We validated the candidate genes through a public database, the database (GSE87433) from the GEO (https://www.ncbi.nlm.nih.gov/geo/query/acc.cgi?acc=GSE87433). The GSE87433 provided by Friedrichs, which included 6 normal control samples and 6 diabetic retinopathy samples in mouse model.

### Statistical analysis

The two-tailed Students’ *t*-test was used to identify the differences between groups. We used the R statistical analysis package (version 3.5.3) to statistically analyze the data. An alpha value of *P* < 0.05 was measured and considered a statistically significant event or comparison in this study.

## Results

### DR microarray datasets

**Figure 1:**
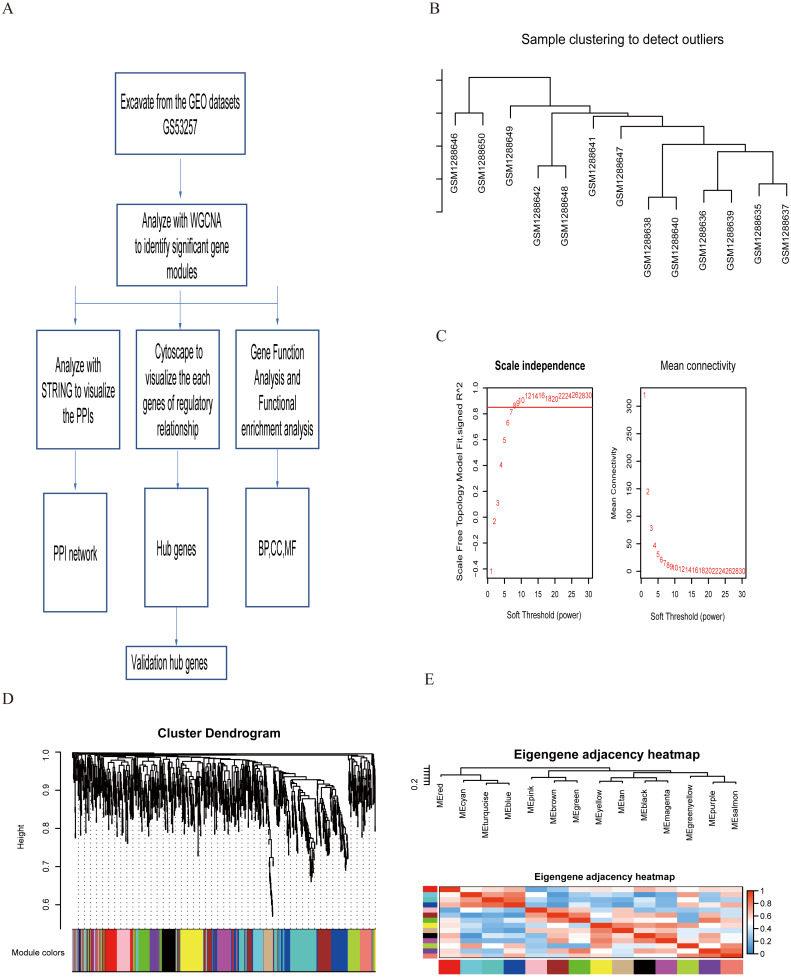
Study flowchart and WGCNA analysis. Details of the study flowchart (A). Dendrogram of sample clustering to detect outliers (B). Power value for the adjacency matrix in WGCNA, where the red line signals 0.9 on the vertical axis (C) The hierarchical clustering dendrogram of genes in GSE with each branch representing a gene, and each color representing a co-expression module in DR (D). The eigengene adjacency heatmap between modules (E).

To establish a gene co-expression network, the raw GSE data was downloaded from GEO. The original data was pre-processed with R for background correlation and standardization. The R-package annotation was used to match the probe of the gene symbol. Probe matching to multiple genes was removed, and the maximal value of a gene that matched multiple probes was taken as the final expression value. One dataset from the GPL18056 platform was selected. Details of the datasets are described in Fig. 1.

### Construction of a co-expression and a PPI network

The clustering tree found that three samples were mixed and eliminated. The remaining 13 samples are analyzed by WGCNA for the next step. We filtered out the probe sets with no significant variance in expression for all analyzed samples (Fig. 1B). Then, the R package WGCNA was used to generate 15 modules from 1,037 probe sets (Figs. 1C and 1D). The black module contained 58 genes, the blue module contained 148 genes, the cyan module contained 42 genes, the pink module contained 53 genes, the green module contained 66 genes, the brown module contained 116 genes, the red module contained 64 genes, the turquoise module contained 149 genes, the yellow module contained 80 genes, the tan module contained 43 genes, the magenta module contained 53 genes, the green/yellow module contained 48 genes, the purple module contained 51 genes, and the salmon module contained 43 genes (Table S2). Genes could not be contained in any of the modules that were otherwise placed into the grey modules and were removed for subsequent analysis (Fig. 1E).

**Figure 2:**
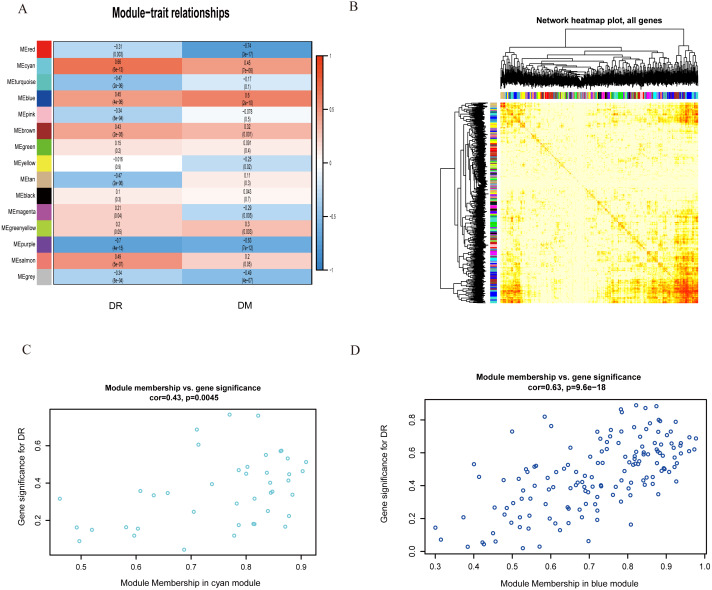
Correlation matrix of the obtained module epigenetic values obtained 15 modules were identified. Within each cell, the upper values represent correlation coefficients between the module eigengene and the trait, and the lower values are the corresponding *P* values. Each of the modules was labeled with a unique color as an identifier. Red indicates positive correlation whereas blue indicates negative correlation. Fifteen modules were identified. (A) The network heat map plot of the co-expressed gene module. (B) Module membership in the cyan module (C) and the blue module (D), showing closely significant intra-modular and gene connectivity.

**Figure 3:**
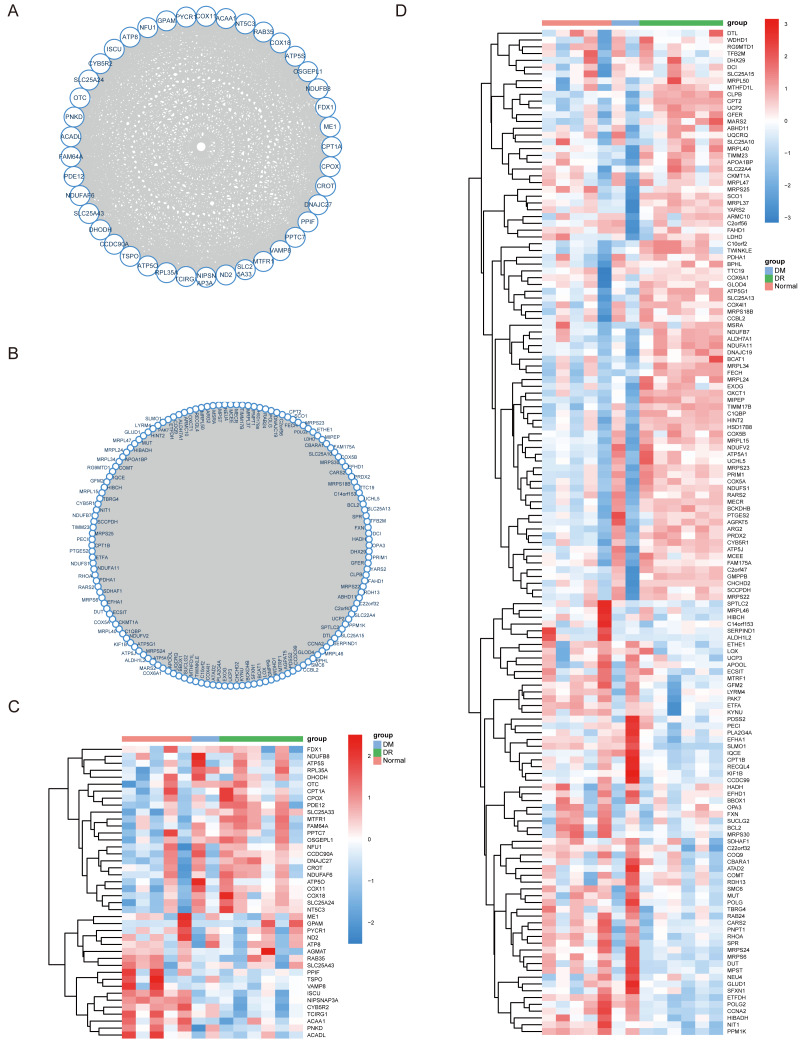
Coding the non-coding gene co-expression (CNC) network for genes. Coding the non-coding gene co-expression (CNC) network for genes grouped in the cyan (A) and blue module (B). Heatmap of genes related to visual perception in cyan module (C) and blue module (D).

Among the 15 modules, the module eigengene (ME) cyan (*r* = 0.66, P = 6E-13) and the ME blue (*r* = 0.6, P = 2E-10) modules were significantly associated with the DR and DM modules (Figs. 2A and 2B). The key drivers in the two modules of interest showed closely significant intramodular and genetic connectivity (Figs. 2C and 2D). Hence, we selected the 42 genes in the ME cyan module and the 149 genes in the ME blue module to structure the PPI and co-expression networks. Finally, a co-expression network containing 41 nodes and 591 edges in the ME cyan module, and 148 nodes and 7,323 edges in the ME blue module were completed (Figs. 3A–3D).

### Pathway and process enrichment analysis

**Figure 4:**
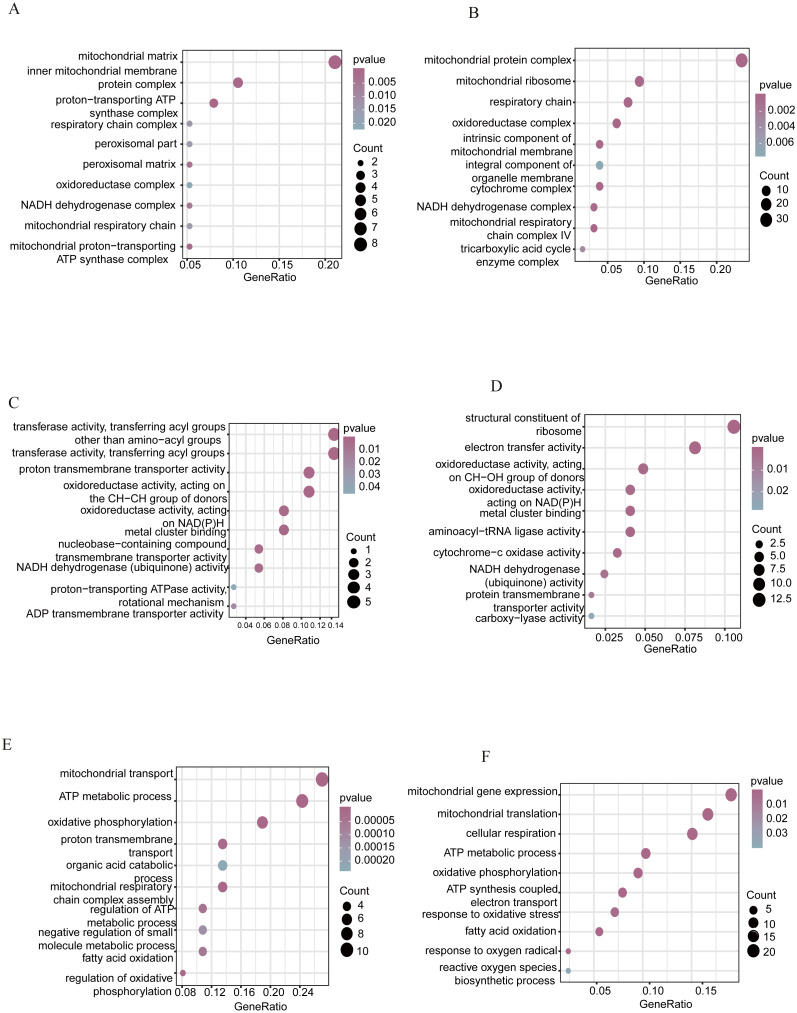
GO enrichment in the CC with the selected 10 terms. GO enrichment in the CC with the selected 10 terms in the cyan (A) and blue module (B). GO enrichment in MF with the selected 10 terms in cyan module (C) and blue module (D). GO enrichment in BP with the selected 10 terms in cyan module (E) and blue module (F).Vertical axis represents GO terms. Circle size represents the number of genes significantly enriched in each GO term. *P* values are marked in different colors. BP, biological process; CC, cellular component; GO, gene ontology; MF, molecular function.

We listed the analysis of the functional enrichment results of two major co-expression modules (Table 1). In the GO cellular component in the cyan module, the most enriched were the following: the mitochondrial matrix, the proton-transporting ATP synthase complex, the NADH dehydrogenase complex, and the oxidoreductase complex, among others (Fig. 4A, Table S3). In the GO cellular component in the blue module, the most enriched were the mitochondrial protein complex, the oxidoreductase complex, and the NADH dehydrogenase complex, among some others (Fig. 4B, Table S4).

**Table 1:** Information of 10 key genes.

**Gene symbol**	**Gene title**	**Related biological process**
SLC25A33	Solute Carrier Family 25 Member 33	GO:0015218: pyrimidine nucleotide transmembrane transporter activity GO:0072531: pyrimidine-containing compound transmembrane transport
MRPS23	Mitochondrial Ribosomal Protein S23	GO:0003723: RNA binding GO:0005515: protein binding GO:0032543: mitochondrial translation
CYB5R1	Cytochrome B5 Reductase 1	GO:0004128: cytochrome-b5 reductase activity, acting on NAD(P)H GO:0016491: oxidoreductase activity GO:0006839: mitochondrial transport
NDUFS1	NADH:Ubiquinone Oxidoreductase Core Subunit S1	GO:0008137: NADH dehydrogenase (ubiquinone) activity GO:0016491: oxidoreductase activity GO:0042775: mitochondrial ATP synthesis coupled electron transport
MECR	Mitochondrial Trans-2- Enoyl-CoA Reductase	GO:0006629: lipid metabolic process GO:0006631: fatty acid metabolic process GO:0055114: oxidation–reduction process
MRPL15	Mitochondrial Ribosomal Protein L15	GO:0070125: mitochondrial translational elongation GO:0070126: mitochondrial translational termination GO:0140053: mitochondrial gene expression
ATP5O	ATP synthase	GO:0046034: ATP metabolic process
MTFR1	Mitochondrial Fission Regulator 1	GO:0005739: mitochondrion GO:0015980: energy derivation by oxidation of organic compounds
CCDC90A	Coiled-Coil Domain- Containing Protein 90A, Mitochondrial	GO:0006816: calcium ion transport GO:0036444: calcium import into the mitochondrion GO:0051561: positive regulation of mitochondrial calcium ion concentration
ACADL	Acyl-CoA Dehydrogenase Long Chain	GO:0000062: fatty-acyl-CoA binding GO:0016401: palmitoyl-CoA oxidase activity GO:0016491: oxidoreductase activity

In the GO molecular function in the cyan module, the most enriched included the oxido-reductase activity, acting on the CH-CH group of donors, the oxidoreductase activity, acting on NAD(P)H, and the ADP transmembrane transporter activity, among others (Fig. 4C, Table S5). Moreover, the blue module was enriched into a molecular function that was involved in multiple fields, including the structural constituent of the ribosome, aminoacyl-tRNA ligase activity, oxidoreductase activity, NADH dehydrogenase (ubiquinone) activity, and oxidoreductase activity (Fig. 4D, Table S6).

The genes of the cyan module were significantly enriched in those exhibiting a biological function with key roles in the following functional domains: mitochondrial transport; ATP metabolic processes; oxidative phosphorylation; fatty acid oxidation; and other functions (Fig. 4E, Table S7). Amongst the GO biological processes in the blue module, the most outstanding genes that were evidently presented included mitochondrial gene expression, oxidative phosphorylation, ATP metabolic processes, fatty acid oxidation, and reactive oxygen species biosynthetic processes, and so on (Fig. 4F, Table S8).

### Identify and verified hub genes in the cyan and the blue modules

**Figure 5:**
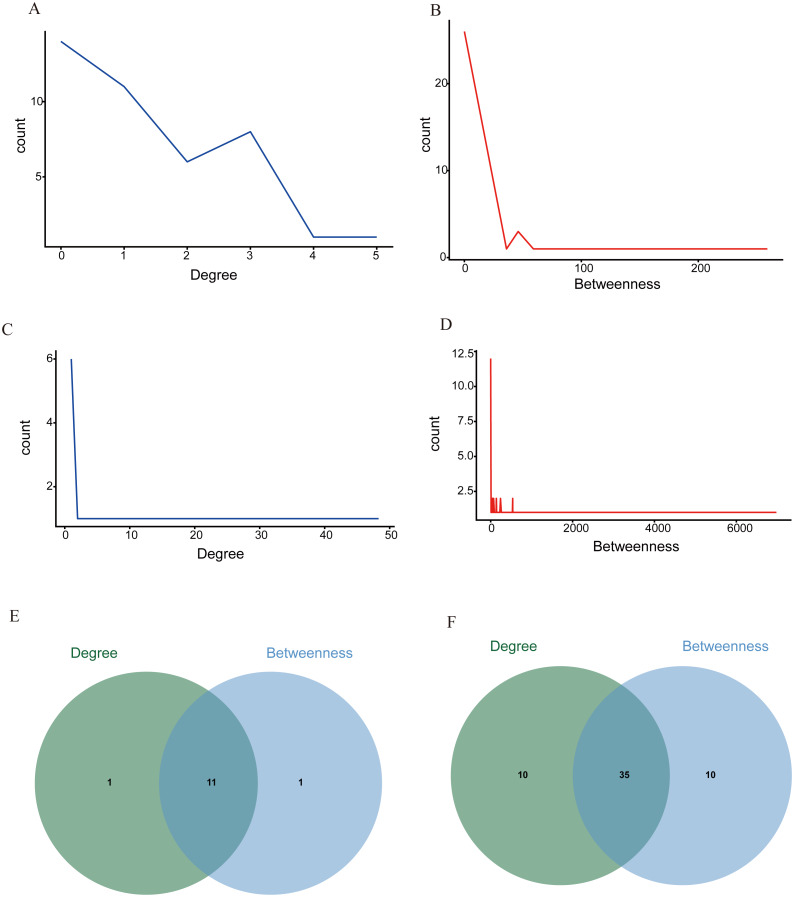
The distribution of the degree of centrality. The distribution of the degree of centrality in the cyan (A) and blue module (C), and the distribution of “betweenness” centrality in the cyan (B) and blue module (D). The intersection of the top 30% molecules in degree centrality and betweenness centrality by Venny 2.1.0 in cyan module (E) and in blue module (F). Results showed 11 hub genes in cyan module and 35 hub genes in blue module were chosen for further study because of their high degree, betweenness values.

**Figure 6:**
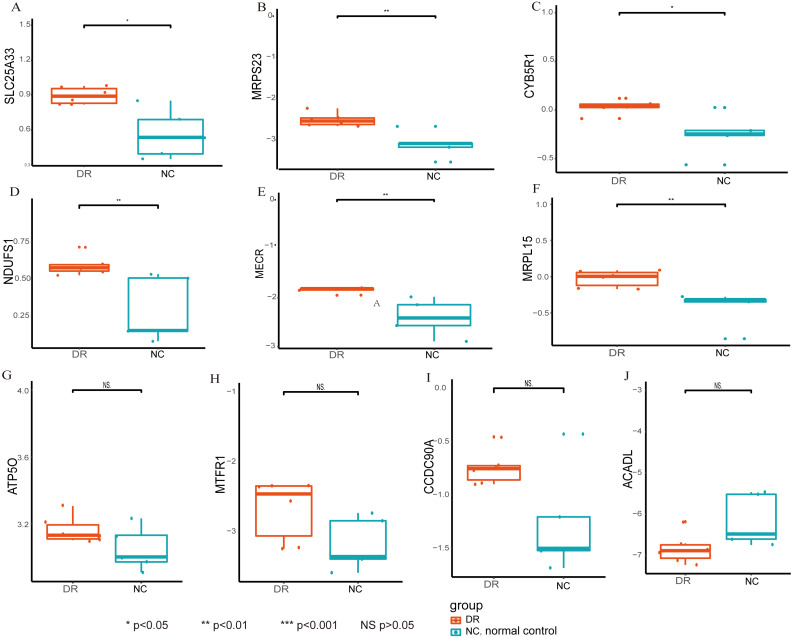
Compared with the normal group, mitochondria-related genes. Mitochondria-related genes expression. Compared the normal group and the DR group,, mitochondria-related genes (SLC25A33 (A), MRPS23 (B), CYB5R1 (C), NDUFS1 (D), MECR (E), MRPL15 (F) were significantly upregulated in the DR group. ^∗^*p* < 0.05, ^∗∗^*p* < 0.01, ^∗∗∗^*p* < 0.001, NS *p* > 0.05.

**Figure 7:**
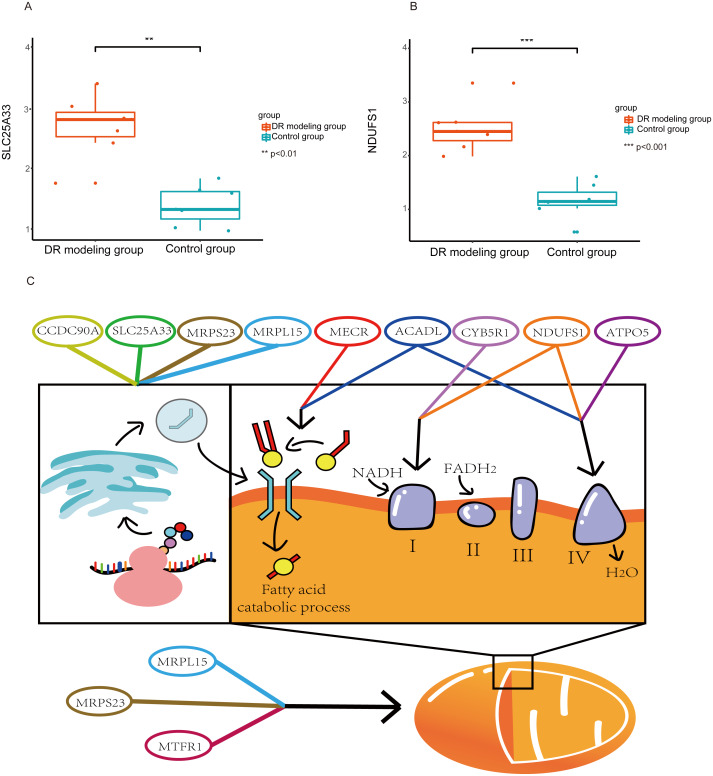
The results of qPCR analysis. The results of qPCR analysis in DR rats model. SLC25A33 (A) and NDUFS1 (B) were highly significant in DR rats modeling group. ^∗∗^*p* < 0.01, ^∗∗∗^*p* < 0.001. Mechanism of the key genes in DR (C).

In this current work, Cytoscape was used to visualize the cyan and blue modules as networks, from which thirty percent of the genes were selected for further analysis by descending sequenced candidate genes of node degree or “betweenness” into the cytoHubba application. The first five genes in each module were considered hub genes (Table 1), and included SLC25A33, ACADL, ATP50, MTFR1, and CCDC90A in the cyan module and NDUFS1, MECR, MRPL15, MRPS23, CYB5R1, in the blue module (Figs. 5A–5D). Moreover, when compared with the normal group, mitochondria-related genes (*i.e.,* SLC25A33, NDUFS1, MRPS23, CYB5R1, MECR, and MRPL15) were significantly upregulated in the DR group (Fig. 6). Summary of the hub genes and the possible mechanisms in DR were suggested in Fig. 7.

### Validation of the key genes

#### Verification of the performance of DR model

Seven rats were successfully modeled, while another 7 rats were treated with an intraperitoneal injection of normal saline as a control. The fasting plasma glucose (FBG) of rats in control subjects was normal (FBG < 6.1 mmol/L), while the FBG in experimental group were significantly higher levels during the experimental process. There was statistically significant difference (*P* < 0:05). The initial body weight of rats was not significantly different (*P* >  0.05). The weight of control group rats was with the steady increase, while the weight in experimental group decreased. The difference was statistically significant between the two groups (*P* < 0:05, Table S9).

SLC25A33 and NDUFS1 were validated by qPCR. The results of qPCR revealed that SLC25A33 and NDUFS1 were significantly differentially expressed between DR model and control group (Figs. 7A and 7B).

### The conservation analysis of SLC25A33 and NDUFS1 genes

To detect the gene conservation of SLC25A33 and NDUFS1 in human, mouse and rat, NCBI database was used. Among the SLC25A33 and NDUFS1 genes, results showed that the gene structure exhibitted high conservatism in human, mouse, and rat (Fig. S1).

### Verification of the external dataset

We found 5 genes annotated in the validation of dataset (GSE87433) on account of the probe sets in mouse model, meanwhile the 5 genes represented the same trend of expression (up-or downregulated) in the microarray analysis (SLC25A33, NDUFS1, ACADL, ATP50, CCDC90A) (Fig. S2), suggesting a good concordance.

## Discussion

DR is a vision-threatening complication of diabetes affecting the structure and cellular composition of the microvasculature. The pathogenesis of DR is complicated and remains largely unclear. It is believed that genetic, environmental and biochemical contribute to the the development of DR. Although some other genes have been reported in DR, the comprehensive analysis of the gene networks and funtional studies associated with the etiology of DR are still lag behind and not been clearly defined.

In our WGCNA analysis, the genes were classified into 15 co-expressed biologically functional modules. This line of study indicated particular novel insights into the pathogenesis of DR at a systems level. In this study, to further understand the significance of these functional modules in the pathogenesis of DR, we performing the enrichment analysis.

Crucial pathways in important modules might perhaps hold the most challenging correlation with the symptoms or pathophysiology of DR. The gene enrichment analysis in the blue module mainly involved those of the “reactive oxygen species biosynthetic and metabolic process, oxidative stress, mitochondrial gene expression and function,” which are relevant to oxidative stress and reactive oxygen responses. Currently, tissue oxidative stress is considered as a vital component in the development of DR (Wu, Tang & Chen, 2014). The enriched function of cyan module pathways mainly contain pathway-specific gene sets involved in the ATP metabolic process, oxidative phosphorylation, fatty acid oxidation, mitochondrial process, and the cellular response to oxidative stress. The regulation of oxidative phosphorylation and ATP metabolic processes suggest that various pathways and metabolism are active in tissue cells when DR is activated.

Therefore, mitochondrial ATP, the response to reactive oxygen species and the response to oxidative stress pathway plays a vital role in the incidence of the pathway leading to, or associated with DR.

However, when considering the molecular mechanisms, and the potential roles played by mitochondrial transport, we recognize that the mitochondrial respiratory chain complex assembly in DR remains poorly understood and warrants further elucidation in the future.

Meanwhile, we listed top 11 related genes in cyan module and 35 related genes in blue module in the visualization operation to show the reliability of the results. Quite a few crucially up-regulated or down-regulated genes were identified in our study, some of which are novel DR gene signatures and their molecular mechanisms and physiological roles still remain largely unknown in DR pathogenesis.

Our discussion therefore mainly focuses on the genes that are selected to be closely related to the occurrence and development of DR. Herein, we further discussed 10 key genes that included: NDUFS1, SLC25A33, MECR, MRPL15, MRPS23, CYB5R1, CCDC90A , MTFR1, ATP50 and ACADL.

Complex I (CI) is the first enzyme of the mitochondrial respiratory chain (Ni et al., 2019). NDUFS1, which belongs to the 75 kDa complex I subunit family located at the mitochondrial inner membrane, is an integral part of the carbohydrate, energy, and amino acid metabolic pathways that play an important role in glycol-metabolic diseases, which have already been reported in many diseases, such as in clear-cell renal-cell carcinoma (Ellinger et al., 2017), lung cancer (Su et al., 2016), schizophrenia and negative symptoms (Zhu et al., 2015), and diabetes mellitus (Wu et al., 2017), but the molecular mechanism underlying its effects remains unclear in diabetes mellitus and also not was not reported in diabetic retinopathy. Moreover, NDUFS1 belongs to oxidative phosphorylation genes, and encodes NADH dehydrogenase with a coordinated increase expression involved in the mitochondrial respiration chain (Liu et al., 2015). However, high glucose conditions induced a significant increase in intracellular reactive oxygen species and subsequently increased the activity of NADH oxidase (Fan, Qiao & Tang, 2017). The biallelic mutations in NDUFS1 dampen the stability of the entire N-module of Complex I, indicative of ROS stress (Ni et al., 2019). In addition, by sequestering NDUFS1, where in super-complex destabilization and oxidative phosphorylation is inefficient (Elkholi et al., 2019). When NDUFS1 is negatively regulated, it results in decreased mitochondrial respiration, and commitment to the mitochondrial apoptotic pathway. Oxidative stress and mitochondrial dysfunction were involved in the pathogenesis of diabetic retinopathy (Wu et al., 2018), we hypothesized that NDUFS1 might play an important role in DR. SLC25A33 belongs to the SLC25 family of mitochondrial carrier proteins that transport molecules over the mitochondrial membrane (Haitina et al., 2006). Previously published work suggested that SLC25A33 promotes cell growth as a mitochondrial UTP carrier (Lyons et al., 2017) and plays a key physiological role in completing transport stages that are vital for mitochondrial DNA and RNA synthesis and breakdown (Di Noia et al., 2014). MECR, a novel gene of mitochondrial and an oxidoreductase, it catalyzes the last step in mitochondrial fatty acid synthesis. In the livers of mice with liver fibrosis, MECR as lipoic acid synthetic pathway enzymes were significantly reduced (Luo & Shen, 2020). Moreover, MECR is reportedly crucial in many diseases, such as hepatocelluar carcinoma (Cai et al., 2019), childhood-onset dystonia and optic atrophy (Heimer et al., 2016). Meanwhile, a marked body of evidence from both cohort and case-control studies have indicated the significant relevance between the disorder of lipid levels and DR (Das et al., 2015). Our results show that MECR was up-regulated in the DR process. This gives a hint that MECR might be vital in the pathogenesis of DR and might also be a novel gene therapeutic target in the treatment of DR. Both MRPL15 and MRPS23 belong to the mitochondrial biomarker set of genes, which might encode mammalian mitochondrial ribosomal proteins and thus assist in protein synthesis within the mitochondrion. Previous research has shown that both MRPL15 and MRPS23 can be selected as companion diagnostics, to decide which breast cancer patients might benefit most from clinical therapy (Sotgia, Fiorillo & Lisanti, 2017). CYB5R1 includes oxidoreductase activity and cytochrome-b5 reductase activity that acts on NAD(P)H. CYB5R1 is important in lipid synthesis in adipocyte mitochondria (Neve et al., 2012), which is a novel beneficial target of demethylation drugs that were also confirmed by real-time RT-PCR (Ning et al., 2017). CCDC90A, also known and renamed as MCUR1, modulates mitochondrial calcium uptake as a regulator of the mitochondrial calcium uniporter complex and thereby maintains normal cellular bioenergetics (Mallilankaraman et al., 2012). Adisorder in CCDC90A can alter the mitochondrial membrane potential and mitochondrial calcium uptake capacity, and can disrupt oxidative phosphorylation, lower cellular ATP and activate AMP kinase-dependent pro-survival autophagy(Mallilankaraman et al., 2012; Paupe et al., 2015). MCUR1 facilitates epithelial-mesenchymal transition and metastasis in hepatocellular carcinoma involved *via* the mitochondrial calcium dependent ROS/Nrf2/Notch pathway (Jin et al., 2019), however, no relevant literature has been reported to date in diabetic retinopathy. ATP50 is a component of the F-type ATPase that is initiated in the mitochondrial matrix, which is involved in oxidative phosphorylation that participates in ATP generation (Rönn et al., 2009). We hypothesized that ATP5O might contribute to DR, and do so by defective ATP production. MTFR1, a target gene of miR-324-5p (Ye et al., 2019), encodes a mitochondrial protein that can promote mitochondrial fission and is crucially involved in oxidative stress. Suppressing MTFR1 translation attenuates mitochondrial fission, apoptosis and myocardial infarction (Wang et al., 2015), which indicates that excessive expression of MTFR1 aggravates mitochondrial fission and apoptosis, meanwhile regulating MTFR1might afford protection against oxidative stress-induced endothelial progenitor cell injury (Chen et al., 2019). Mitochondrial function has a profound effect on DR (Shao et al., 2019), among these influences, MTFR1 in all probability might play a vital role in the mechanism of DR. ACADL belongs to the mitochondrial flavoenzyme family that are commonly involved in fatty acid. Precious studies have reported the involvement of lipids in the progression of DR (Zhou et al., 2018), whereas an increased acetylation of ACADL is related to decreased fat metabolism, which contributes to damaged mitochondrial function and protein acetylation to influence fatty acid oxidation and the development of metabolic dysregulation (Softic et al., 2019). This means hint us that ACADL may be involved in DR, although this hypothesis requires further investigation.

All of the above related experiments of the10 genes are not reported in DR. In terms of validation of the key genes, first, an external validation was carried out using the validation dataset. Last, among the significantly up-regulated genes, the high ranking SLC25A33 and NDUFS1 were validated using RT-qPCR analysis in our experiment. The results revealed SLC25A33 and NDUFS1 expression were highly significant in DR group, which deserved our further attention. Both the genes are associated with mitochondrial function, oxidative stress and fatty acid metabolism. However, further investigations evaluating the specific effects of SLC25A33 and NDUFS1 on DR development and the molecular mechanism underlying its effects are required. Moreover, we analyzed the gene conservation of SLC25A33 and NDUFS1 in human, mouse and rats, and the results showed that the gene structure exhibitted high conservatism among them, which also further proves that our results are reliable.

Our findings have identified and selected underlying genes that might play vital roles in DR pathogenesis. Meanwhile, it highlighted the importance of mitochondria and oxidative stress of the DR, particularly in providing a more in-depth research on molecular level. Nevertheless, we acknowledge that several potential limitations in our study need to be considered. First, original data lacks sufficient clinical data and sample outcomes, which is limited in the judgment of module importance. Second, due to human retinal sample requires the acquisition from human cadaver eye, it is harder to obtain in clinical practice. So we only verify our results using animal models. Meanwhile, the conservation of RNA expression level should be considered. But verify the results *via* a large-scale human retinal sample will be better. Consequently, further specific studies are needed to provide greater insights into DR progression and diagnosis with the aim of strengthening the management of this disease.

## Conclusion

In the present study, the biggest characteristic is that not only novel underlying genes were found *via* bioinformatics analysis, and which were verified by real-time PCR in DR rats modeling and an external database, but also gene co-expression network analysis highlights the importance of mitochondria and oxidative stress in the pathophysiology of diabetic retinopathy. Mitochondria involved in the process of disease initiation and progression still is a hot spot in diabetic retinopathy. Our findings clearly elucidate the potential role of the underlying genes and pathways in the development of DR, and both SLC25A33 and NDUFS1 may serve as potential disease markers and therapeutic target for DR.

## Supplemental Information

10.7717/peerj.12126/supp-1 Supplemental Information 1 The primer sequence in qPCR

10.7717/peerj.12126/supp-2 Supplemental Information 2 The genes in different modules

10.7717/peerj.12126/supp-3 Supplemental Information 3 GO enrichment in the CC with the selected 10 terms in the cyan module

10.7717/peerj.12126/supp-4 Supplemental Information 4 GO enrichment in the CC with the selected 10 terms in the blue module

10.7717/peerj.12126/supp-5 Supplemental Information 5 GO enrichment in the MF with the selected 10 terms in the cyan module

10.7717/peerj.12126/supp-6 Supplemental Information 6 GO enrichment in the MF with the selected 10 terms in the blue module

10.7717/peerj.12126/supp-7 Supplemental Information 7 GO enrichment in the BP with the selected 10 terms in the cyan module

10.7717/peerj.12126/supp-8 Supplemental Information 8 GO enrichment in the BP with the selected 10 terms in the blue module

10.7717/peerj.12126/supp-9 Supplemental Information 9 Comparison of body weight and fasting blood sugar at different time points in rats of the two groups

10.7717/peerj.12126/supp-10 Supplemental Information 10 The result of conservation analysis of SLC25A33 and NDUFS1 genes in human, mouse, and rat The results showed that the gene structure exhibitted high conservatism in human, mouse, and rat.

10.7717/peerj.12126/supp-11 Supplemental Information 11 In mouse model, the 5 genes represented the same trend of expression (up-or downregulated) in the microarray analysis (SLC25A33 (A), NDUFS1 (B), ATP5O (C), CCDC90AD (D), ACADL (E)) in the validation of dataset (GSE87433)

10.7717/peerj.12126/supp-12 Supplemental Information 12 Raw data of qPCR

10.7717/peerj.12126/supp-13 Supplemental Information 13 Raw data figures

10.7717/peerj.12126/supp-14 Supplemental Information 14 ARRIVE Checklist

## Additional Information and Declarations

### Data Availability

The following information was supplied regarding data availability: The raw measurements are available in Supplemental File 1-raw data. The raw data are showing the results of PCR.
