# Supplementary material for: Identification and validation of hub genes for diabetic retinopathy
Source: PeerJ. 2021 Sep 13;9:e12126. doi: 10.7717/peerj.12126 (PMC8445088; doi:10.7717/peerj.12126)
Supplement: Supplemental Information 1 [file peerj-09-12126-s001.docx]

Supplementary Table 1. The primer sequence in qPCR

| Gene symbol | Primer | Sequence 5‘-3‘ |
| --- | --- | --- |
| SLC25A33 | Forward | TGTACTTCGCGTGCTACTCC |
|  | Reverse | GCTTGCAGCCTCTCATCTTTC |
| NDUFS1 | Forward | TTTCTAGAGGGGAAGCGTGC |
|  | Reverse | TGCAGCGGGTACACTGTATG |
| actin | Forward | ACATCCGTAAAGACCTCTATGCC |
|  | Reverse | TACTCCTGCTTGCTGATCCAC |
