# Supplementary material for: Identification and validation of hub genes for diabetic retinopathy
Source: PeerJ. 2021 Sep 13;9:e12126. doi: 10.7717/peerj.12126 (PMC8445088; doi:10.7717/peerj.12126)
Supplement: Supplemental Information 2 [file peerj-09-12126-s002.docx]

**Supplementary Table 2.** The genes in different modules

| Module | Number of genes |
| --- | --- |
| Black module | 58 |
| Blue | 148 |
| Cyan | 42 |
| Pink | 53 |
| Green | 66 |
| Brown | 116 |
| Red | 64 |
| Turquoise | 149 |
| Yellow | 80 |
| Tan | 43 |
| Magenta | 53 |
| Green/yellow | 48 |
| Purple | 51 |
| Salmon | 43 |
| Gray | 23 |
