# Supplementary material for: Identification and validation of hub genes for diabetic retinopathy
Source: PeerJ. 2021 Sep 13;9:e12126. doi: 10.7717/peerj.12126 (PMC8445088; doi:10.7717/peerj.12126)
Supplement: Supplemental Information 3 [file peerj-09-12126-s003.docx]

**Supplementary Table 3.** GO enrichment in the CC with the selected 10 terms in the cyan module.

GO, gene ontology; CC, cellular component.
