# Supplementary material for: Identification and validation of hub genes for diabetic retinopathy
Source: PeerJ. 2021 Sep 13;9:e12126. doi: 10.7717/peerj.12126 (PMC8445088; doi:10.7717/peerj.12126)
Supplement: Supplemental Information 6 [file peerj-09-12126-s006.docx]

**Supplementary Table 6**. GO enrichment in the MF with the selected 10 terms in the blue module.

GO, gene ontology; MF, molecular function.
