# Supplementary material for: Identification and validation of hub genes for diabetic retinopathy
Source: PeerJ. 2021 Sep 13;9:e12126. doi: 10.7717/peerj.12126 (PMC8445088; doi:10.7717/peerj.12126)
Supplement: Supplemental Information 9 [file peerj-09-12126-s009.docx]

**Supplementary Table 9.** Comparison of body weight and fasting blood sugar at different time points in rats of the two groups.

| **General state** |  | **Group Control** | **Group DR** | **p-Value** |
| --- | --- | --- | --- | --- |
| Body weight | 0-week | 337.81±21.29 | 347.71±28.95 | 0.4801 |
|  | 4- week | 348.16±27.50 | 267.51±8.75 | 0.0000084 |
|  | 8- week | 368.49±17.52 | 264.64±8.97 | 3.0546e-8  (< 0.05) |
|  | 12- week | 410.44±13.04 | 259.54±9.12 | 9.7399e-12(< 0.05) |
| Fasting blood glucose | 0-week | 4.44±0.35 | 21.73±0.91 | 5.4521e-15  (< 0.05) |
|  | 4- week | 4.21±0.23 | 24.13±0.94 | 9.5419e-16  (< 0.05) |
|  | 8- week | 4.49± 0.42 | 27.63±2.62 | 2.6237e-11  (< 0.05) |
|  | 12- week | 4.44 ±0.24 | 28.70± 2.91 | 4.7404e-11  (< 0.05) |
