# Supplementary material for: Identification and validation of hub genes for diabetic retinopathy
Source: PeerJ. 2021 Sep 13;9:e12126. doi: 10.7717/peerj.12126 (PMC8445088; doi:10.7717/peerj.12126)

```
HUMAN.seq    ATGTTAAGGATACCTGTAAGAAAGGCCTTAGTAGGCCTTT    40
MOUSE.seq    ATGTTAAGGATACCTATAAAAAGGGCCTTGATAGGCCTTT    40
rat.seq      ATGTTAAGGATACCTGTAAAAAGGGCCTTGATAGGCCTTT    40
Consensus    atgttaaggatacct taa aa ggcctt  taggccttt

HUMAN.seq    CTAAGTCTCCTAAAGGATGTGTTCGAACAACTGCCACAGC    80
MOUSE.seq    CTAATTCTCCTAAAGGATATGTTCGCACAACTGGCACAGC    80
rat.seq      CTAAGTCTCCTAAAGGATATGTTCGATCAACTGGCACAGC    80
Consensus    ctaa tctcctaaaggat tgttcg  caactg cacagc

HUMAN.seq    AGCAAGCAACTTGATTGAAGTATTTGTTGATGGTCAGTCT   120
MOUSE.seq    AGCAAGTAACTTGATTGAAGTATTTGTTGATGGTCAGTCT   120
rat.seq      AGCAAGTAACTTGATTGAAGTATTTGTTGATGGTCAGTCT   120
Consensus    agcaag aacttgattgaagtatttgttgatggtcagtct

HUMAN.seq    GTCATGGTGGAACCGGGAACGACCGTCCTCCAA.......   153
MOUSE.seq    GTCATGGTGGAACCAGGAACCACTGTTCTGCAGGCTTGCG   160
rat.seq      GTCATGGTGGAACCAGGAACCACTGTCCTGCAGGCCTGCG   160
Consensus    gtcatggtggaacc ggaac ac gt ct ca
```

NDUFS1

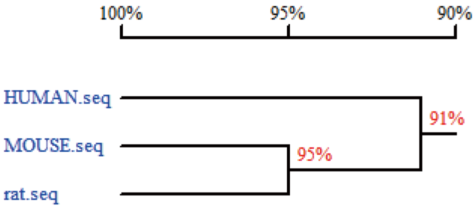


```
HUMAN.seq    ATGGCGACGGGCGGCCAGCAGAAGGAGAACACGCTGCTTC    40
MOUSE.seq    ATGGCGACGGGCACTCAGCAGAAAGAGAACACGCTGCTTC    40
rat.seq      ATGGCGACGGACACTCAGCAGAAGGAGAACACGCTGCTTC    40
Consensus    atggcgacgg c   cagcagaa gagaacacgctgcttc

HUMAN.seq    ACCTCTTCGCCGGCGGGTGTGGAGGCACAGTTGGTGCTAT    80
MOUSE.seq    ATCTCTTCGCCGGCGGGTGCGGGGGCACAGTTGGTGCTAT    80
rat.seq      ATCTCTTCGCCGGCGGGTGTGGAGGTACAGTTGGTGCTAT    80
Consensus    a ctcttcgccggcgggtg gg gg acagttggtgctat

HUMAN.seq    TTTCACTTGTCCACTAGAAGTCATTAAGACACGGTTGCAG   120
MOUSE.seq    TTTCACATGTCCACTAGAAGTCATTAAGACGCGACTGCAG   120
rat.seq      TTTCACATGTCCACTAGAAGTCATTAAGACGAGACTGCAG   120
Consensus    tttcac tgtccactagaagtcattaagac  g  tgcag

HUMAN.seq    TCTTCAAGATTAGCTCTCCGGACAGTCTACTATCCTCAGG   160
MOUSE.seq    TCTTCGAGACTAGCTCTTCGGACGGTGTATTACCCTCAGG   160
rat.seq      TCTTCGAGACTAGCTCTTCGGACGGTGTATTATCCTCAGG   160
Consensus    tcttc aga tagctct cggac gt ta ta cctcagg
```

SLC25A33

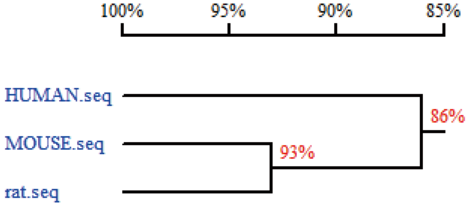

Supplement: Supplemental Information 10 — The results showed that the gene structure exhibitted high conservatism in human, mouse, and rat. [file peerj-09-12126-s010.pdf]
