# Supplementary figures and images for: Identification and validation of hub genes for diabetic retinopathy
Source: PeerJ. 2021 Sep 13;9:e12126. doi: 10.7717/peerj.12126 (PMC8445088; doi:10.7717/peerj.12126)

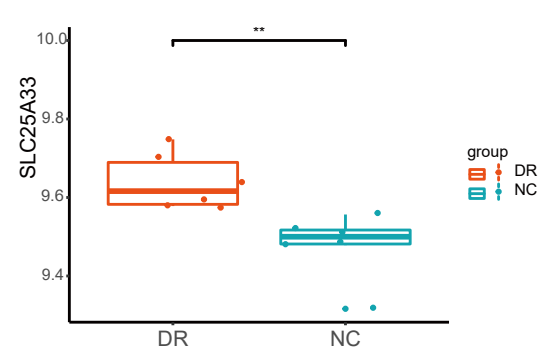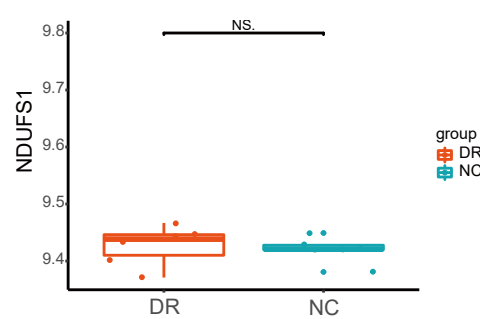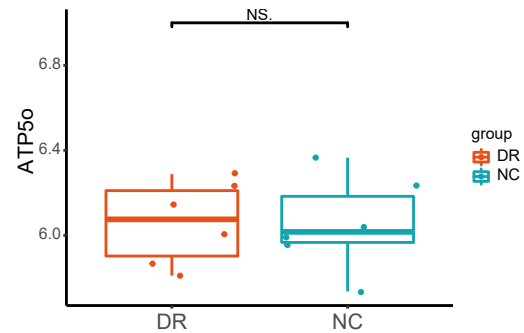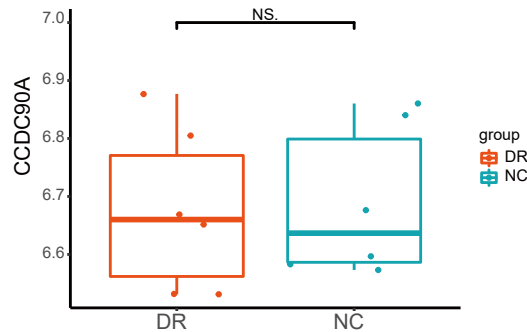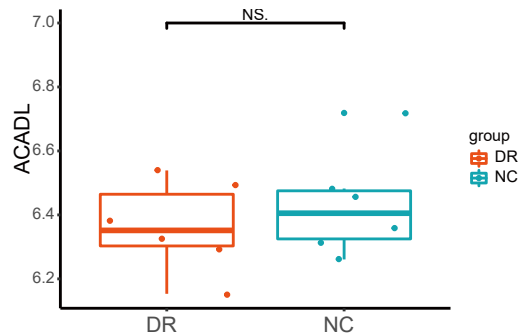

\* p<0.05

\*\* p<0.01

\*\*\* p<0.001

NS p>0.05

Supplement: Supplemental Information 11 [file peerj-09-12126-s011.pdf]

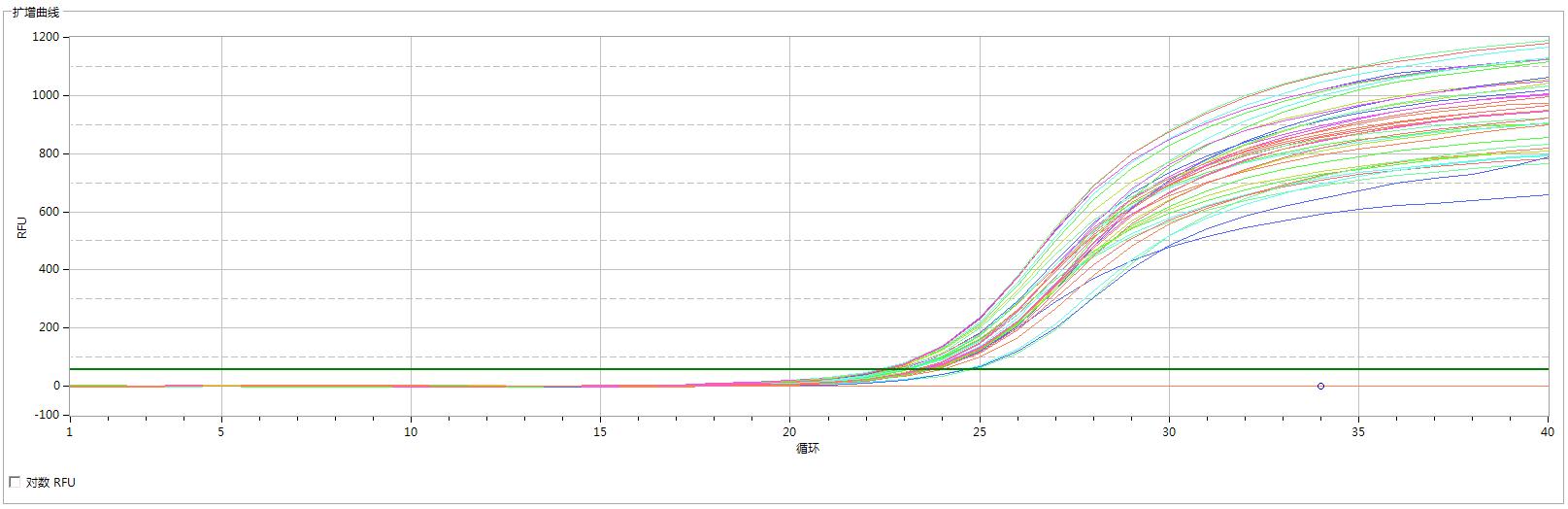

Supplement: Supplemental Information 12 [file peerj-09-12126-s012.zip › files 1-raw data/the melting curve, amplification curve and figure of RNA/NDUFS1 amplification curve.jpg]

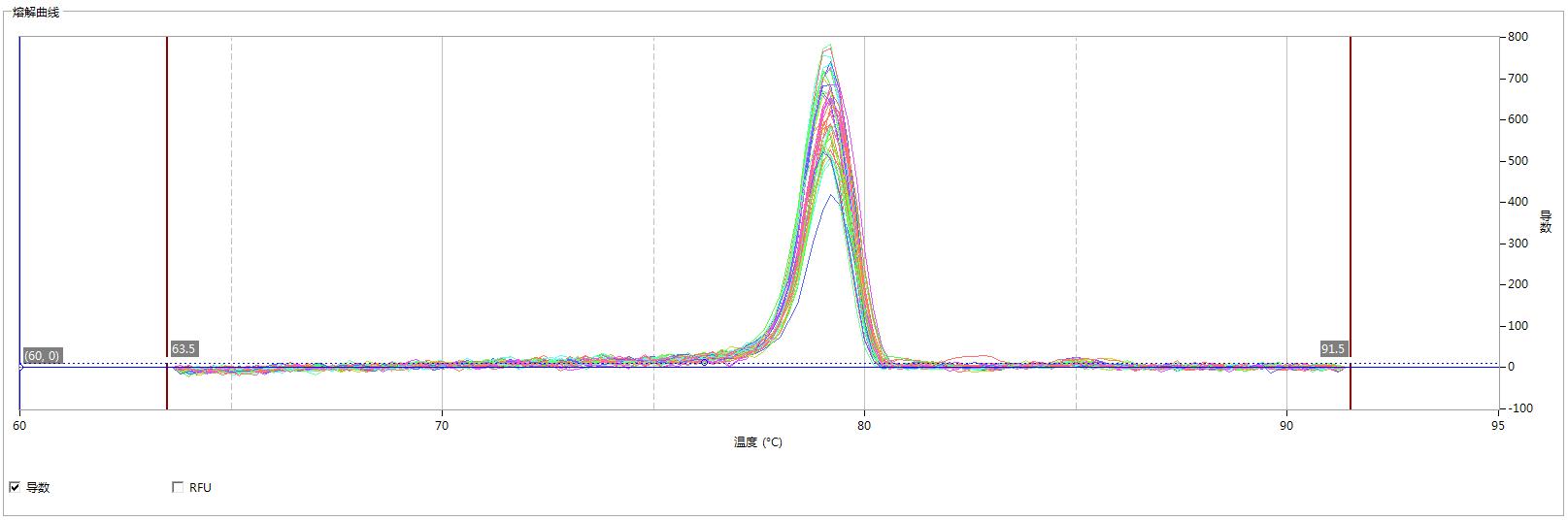

Supplement: Supplemental Information 12 [file peerj-09-12126-s012.zip › files 1-raw data/the melting curve, amplification curve and figure of RNA/NDUFS1 melting curve.jpg]

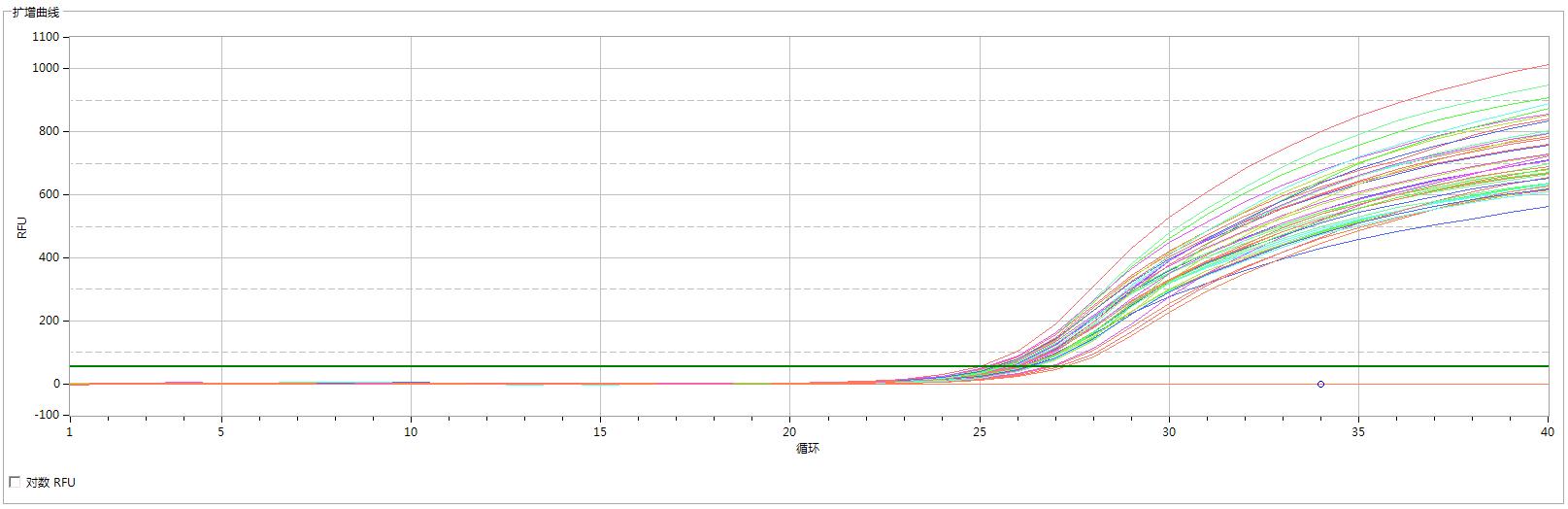

Supplement: Supplemental Information 12 [file peerj-09-12126-s012.zip › files 1-raw data/the melting curve, amplification curve and figure of RNA/SLC25A33 amplification curve.jpg]

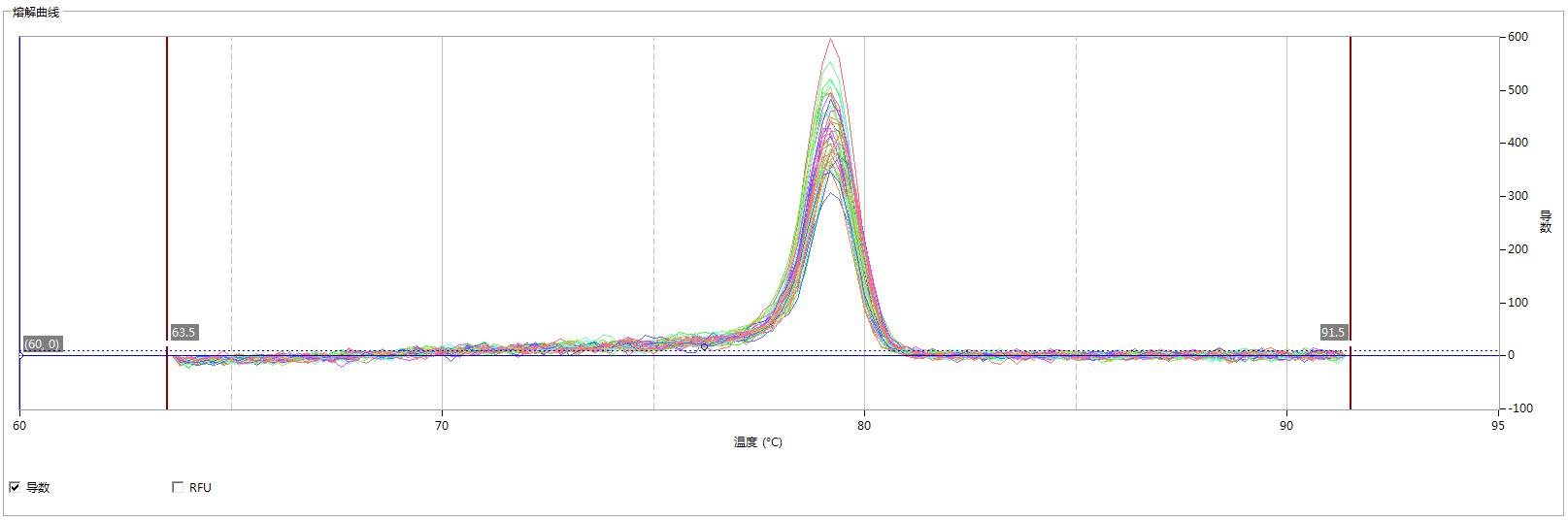

Supplement: Supplemental Information 12 [file peerj-09-12126-s012.zip › files 1-raw data/the melting curve, amplification curve and figure of RNA/SLC25A33 melting curve.jpg]

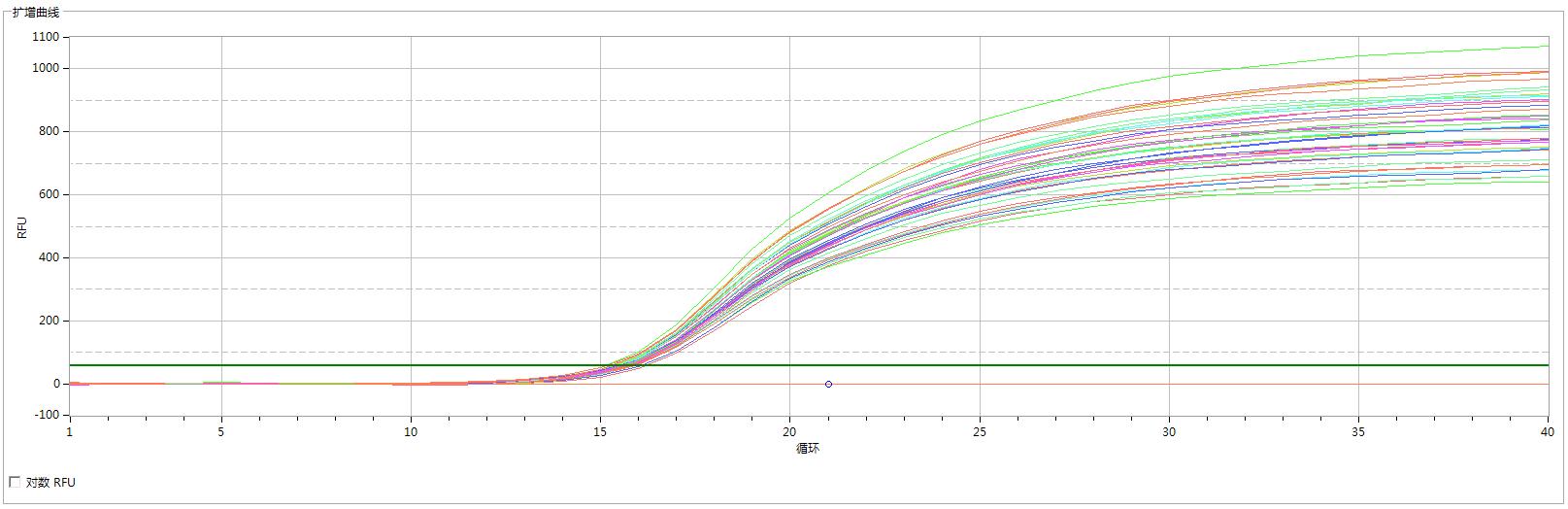

Supplement: Supplemental Information 12 [file peerj-09-12126-s012.zip › files 1-raw data/the melting curve, amplification curve and figure of RNA/actin amplification curve.jpg]

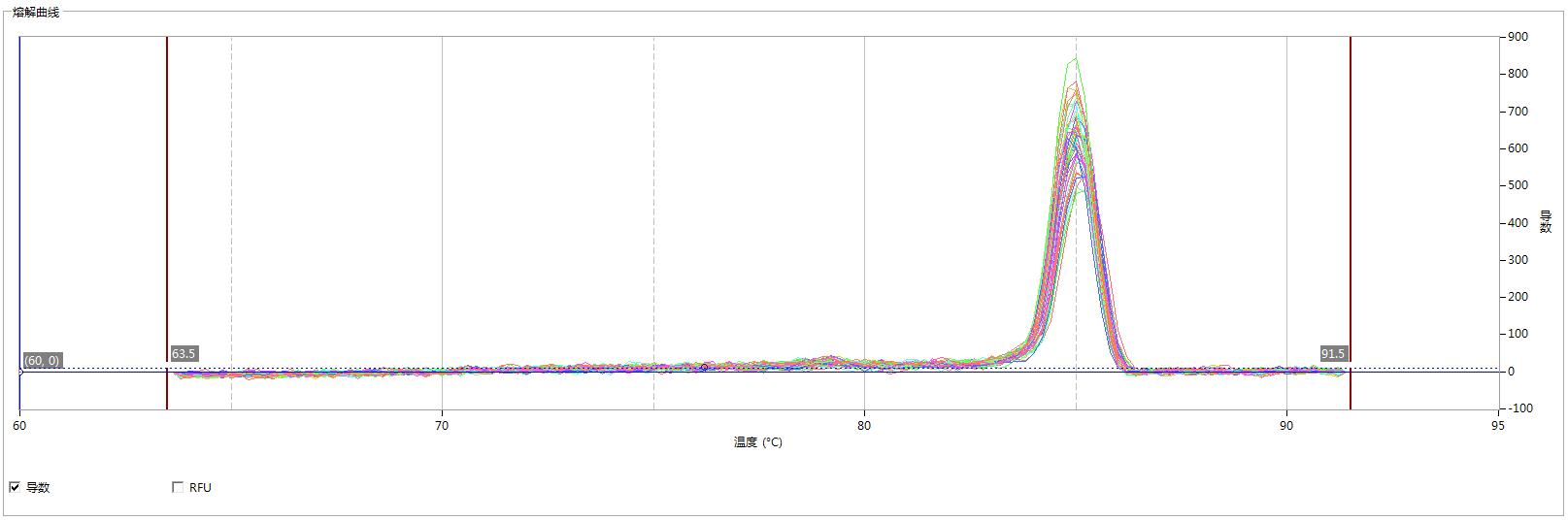

Supplement: Supplemental Information 12 [file peerj-09-12126-s012.zip › files 1-raw data/the melting curve, amplification curve and figure of RNA/actin melting curve.jpg]

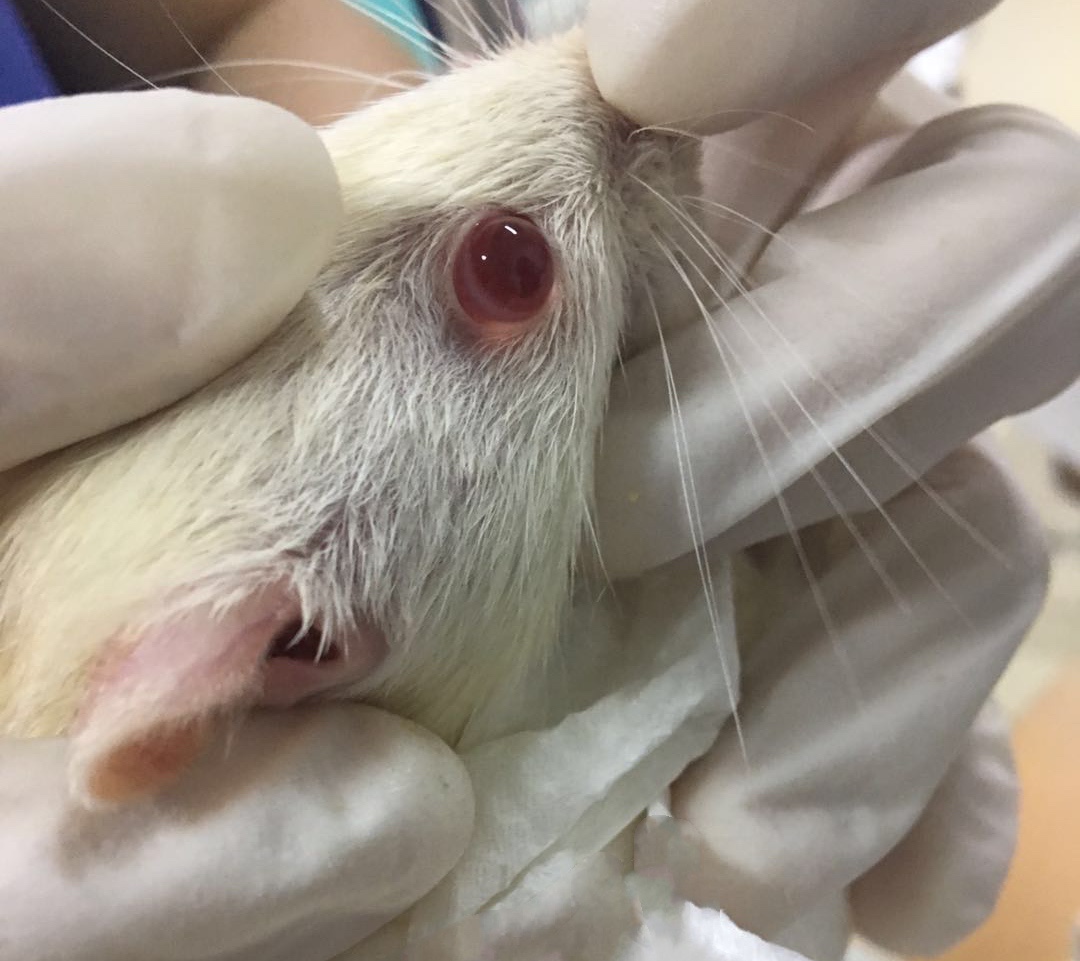

Supplement: Supplemental Information 13 [file peerj-09-12126-s013.zip › files 2-supporting information for experimental manipulation/fig1s-rat.jpg]

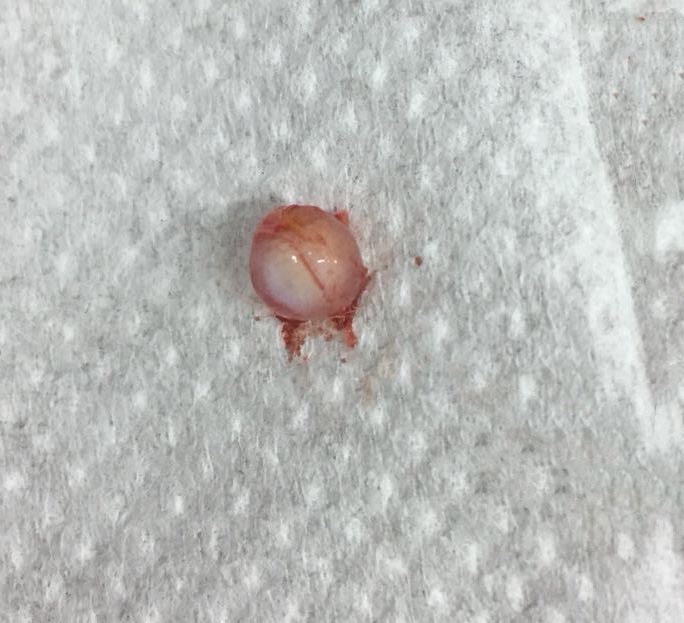

Supplement: Supplemental Information 13 [file peerj-09-12126-s013.zip › files 2-supporting information for experimental manipulation/fig2s-eyeball.jpg]

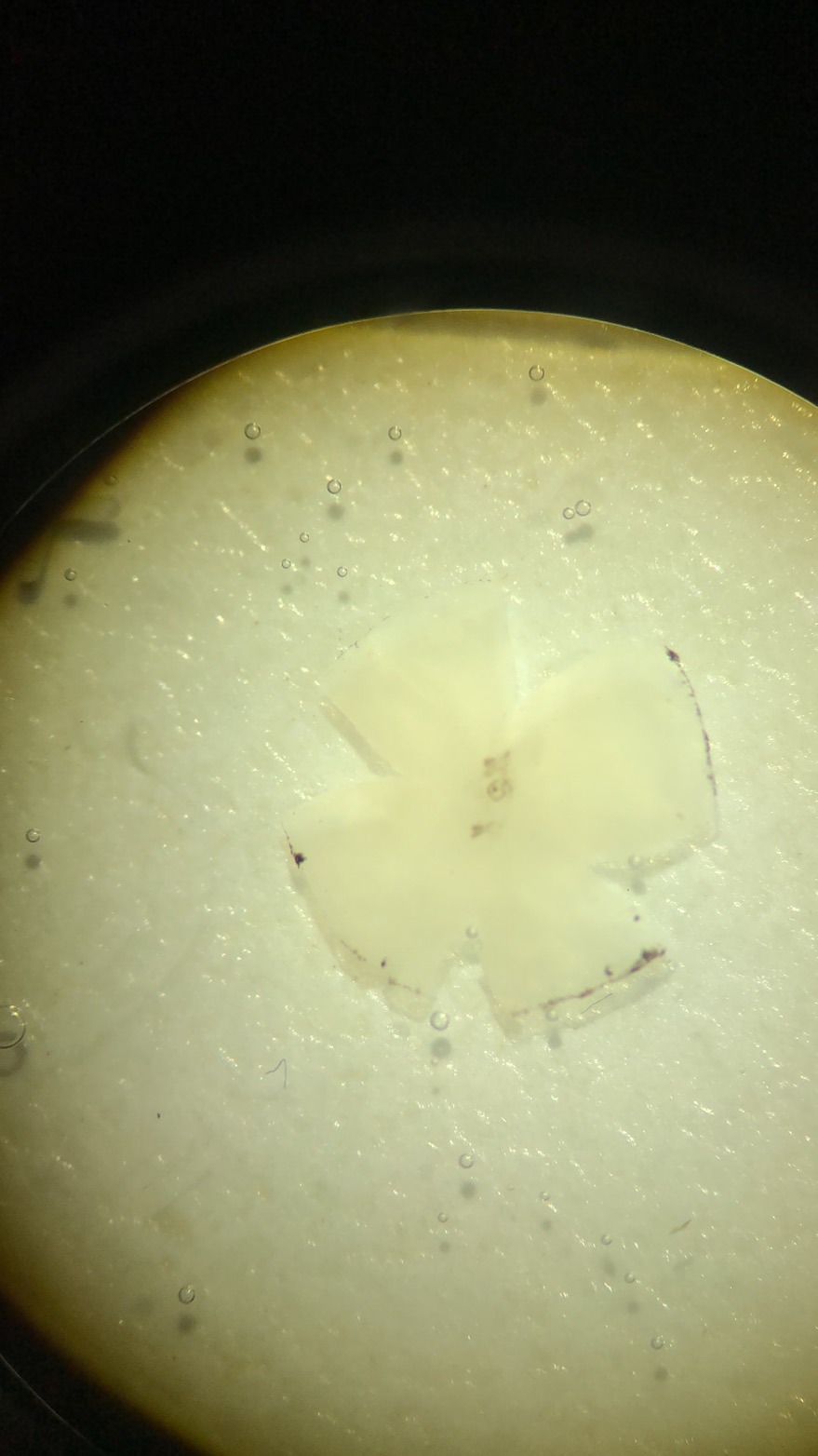

Supplement: Supplemental Information 13 [file peerj-09-12126-s013.zip › files 2-supporting information for experimental manipulation/fig3s-anatomy histology.jpg]
